# Access to primary health care: perspectives of primary care physicians and community stakeholders

**DOI:** 10.1186/s12875-024-02312-9

**Published:** 2024-05-06

**Authors:** Darene Toal-Sullivan, Simone Dahrouge, Johanna Tesfaselassie, Laura Olejnik

**Affiliations:** 1 C.T. Lamont Primary Health Care Research Centre, Bruyere Research Institute, Ottawa, Canada; 2 Faculty of Medicine, University of Ottawa, Ottawa, Canada; 3 Faculty of Science, University of Ottawa, Ottawa, Canada; 4 Hamilton Health Sciences, McMaster University, Hamilton, Canada

**Keywords:** Patient navigation, Community resources, Social determinants of health

## Abstract

**Background:**

Action on the social determinants of health is important to strengthen primary health care and promote access among underserved populations. We report on findings from stakeholder consultations undertaken at one of the Canadian sites of the Innovative Models Promoting Access-to-Care Transformation (IMPACT) program, as part of the development of a best practice intervention to improve access to primary health care. The overarching objective of this qualitative study was to understand the processes, barriers, and facilitators to connect patients to health enabling community resources (HERs) to inform a patient navigation model situated in primary care.

**Methods:**

Focus groups and interviews were conducted with primary care physicians, and community health and social service providers to understand their experiences in supporting patients in reaching HERs. Current gaps in access to primary health care and the potential of patient navigation were also explored. We applied Levesque et al., (2013) access framework to code the data and four themes emerged: (1) Approachability and Ability to Perceive, (2) Acceptability and Ability to Seek, (3) Availability and Accommodation, and Ability to Reach, and (4) Appropriateness.

**Results:**

Determinants of access included patient and provider awareness of HERs, the nature of the patient-provider relationship, funding of HERs, integration of primary and community care services, and continuity of information. Participants’ perspectives about the potential scope and role of a patient navigator provided valuable insight for the development of the Access to Resources in the Community (ARC) navigation model and how it could be embedded in a primary care setting.

**Conclusion:**

Additional consultation with key stakeholders in the health region is needed to gain a broader understanding of the challenges in caring for primary care patients with social barriers and how to support them in accessing community-based primary health care to inform the design of the ARC intervention.

**Supplementary Information:**

The online version contains supplementary material available at 10.1186/s12875-024-02312-9.

## Introduction

### Access to primary health care

In Canada, patients’ first point of contact with the health care system is through primary care delivered by a family physician, or nurse practitioner or other health care provider [1]. Primary care provides person-focused care over a longitudinal course, working with patients, families, and communities, to coordinate and provide continuity across the continuum of care [2]. Primary health care adopts a broader multisystem approach and considers the range of social, economic and environmental conditions that influence the health of individuals and populations [3]. While equitable access is a core principle of primary health care [4] many barriers including gender, race, education, mental health, co-morbidities, socioeconomic status, and housing affect the most vulnerable populations and those at greatest need for care [3, 5–7]. Access to care and factors that influence access for different populations, health needs and for diverse contexts is complex and researchers have proposed different frameworks to conceptualize this interaction between individuals, and health and social systems [8–10]. Levesque et al., [8] conceived access to care as multidimensional involving the interaction between the characteristics of health care providers, services, and systems; and the abilities and social determinants of individuals and populations. There are five dimensions of access that pertain to available healthcare services and systems: (1) approachability, (2) acceptability, (3) availability and accommodation, (4) affordability, and (5) appropriateness. These dimensions correspond to the following individuals’ abilities which vary based on knowledge, health beliefs, culture, and social determinants: (1) ability to perceive the need for care, (2) ability to seek care, (3) ability to reach care, (4) ability to pay for care, and (5) ability to engage in care. These dimensions are depicted on a continuum of access from the early stages such as understanding one’s health care needs, perceiving that there is care available, and seeking that care, to later stages of access such as obtaining an appointment, paying for services, and receiving appropriate care. Access is realized when barriers are overcome at each stage of the continuum.

There is increased recognition of the need to target the social determinants of health to reduce health inequalities among underserved populations [11, 12]. While the literature often discusses barriers and facilitators to access at a community, population [13, 14] and policy level [6], further research is needed about the perspective of primary care physicians and community service providers’ in promoting access to primary health care [15, 16]. Primary care providers (PCPs) are ideally situated in the healthcare system to target the social determinants of health and integrate various aspects of patient care including individual factors (personal health practices, gender, education) and contextual factors (social, economic, and material resources) that influence health [17–19]. Research indicates however that PCPs face various barriers to addressing these determinants in clinical practice including lack of confidence in meeting patients’ social needs, limited awareness of local community resources, and lack of consultation time [20]. This influences providers’ ability to adopt a holistic approach to person-centered care including eliciting patients’ concerns and addressing their social challenges [16, 21–23].

The work reported here was conducted as part of Innovative Models Promoting Access-to-Care Transformation (IMPACT), a 5-year Canadian-Australian research program that aimed to identify, implement and trial best practice interventions to improve access to primary healthcare for vulnerable populations [6]. There were 3 Canadian (Ontario, Alberta and Quebec) and 3 Australian sites under the IMPACT program. The Champlain Health Region in Ontario, one of the Canadian IMPACT sites, sought to establish an innovation that would help individuals overcome barriers to access health and social resources. An extensive phase of consultations with primary care physicians and community stakeholders informed the development of the Access to Resources in the Community (ARC), an innovative navigation model situated in primary care. The objectives of this study were threefold: (1) to understand primary care physicians and community stakeholders’ awareness and use of health enabling community resources (HERs), (2) to identify perceived barriers and facilitators to access HERs, and (3) to develop recommendations to support patients in reaching HERs for their health and well-being.

## Methods

### Study design and participants

A case study methodology provided an in-depth exploration of the multi-faceted and complex issue or case of access to care with its unique challenges and opportunities within particular community, the Champlain Health Region [24–26]. A post-positivist paradigm was used to understand how participants from the community experienced and understood access to care. Data were collected using two methods: (1) focus groups comprising different stakeholders, and (2) interviews with primary care physicians (PCPs). A purposeful selection strategy was used to recruit participants. PCPs in the Champlain Health Region known to have an interest in primary health care research and equitable access were recruited by telephone and email for interviews. The Champlain Health Region is an area defined by the provincial ministry of health for the planning and delivery of primary care services in eastern Ontario, Canada. The study sites were located in eastern Ontario. The socio-demographic profile of the population in Champlain region is presented in Table 1. Stakeholders known to the ARC Advisory Committee, with interest or experience in navigation as providers or recipients of primary health care services were recruited by email for the focus groups. A focus group topic guide was developed from a literature review about organizational interventions to improve primary care access, the critical factors in primary care referral to community services, and patient navigation. An abbreviated version of the focus group questions can be found in Appendix A. The interview guide was developed based on findings from four of the focus groups conducted for this study and a review of the literature on access to primary care by vulnerable populations. An abbreviated version of the interview guide can be found in Appendix B.

**Table 1:** Profile of the population demographics in the Champlain Health Region

Variable	Champlain LHIN
Total population (census 2016), n	1,292,639
15 to 64 years %	66.8
Speak French only %	1.9
Male sex (census 2016) %	48.8
Unemployment %	7.1
Immigrants %	18.7
Prevalence of low income based on the Low-income cut-offs, after tax (LICO-AT) (%)	9.1
No certificate, diploma or degree (15 years and above, living in private households %)	14.3

(Census profile, 2021 Census of population) [42]

### Data collection

A brief description of study participants can be found in Table 2. Six focus groups approximately 1.5 h in duration were conducted with a total of 40 participants. Participants included mental Health System Clients and Caregivers (an identified priority in the region), CHCs (Urban and rural) who have vast experience in supporting patients with higher vulnerability and complex patients, multicultural Health Navigators and patients and primary care practice members from the participating practices to learn from their lived experience in primary care practice. Discussion was facilitated by the principal investigator and a research associate. The principal investigator was the content expert who also leads the Access to Resources in the Community program. The research associate had experience in qualitative research, community-based evaluation and research for non-profit organisation, focused on under-served populations, as well as patient engagement. Individual interviews with six PCPs were carried out, with a duration ranging from twenty minutes to 1 h. Interviews were conducted by two members of the ARC research team. Five of the interviews were conducted in person in a non-clinical setting, while one interview was conducted by telephone.

**Table 2:** Study participants

Focus group	Participants
**Focus Group Participants**	
FG1: Mental Health System Clients and Caregivers	4 participants (1 family member of a person with schizophrenia, 1 mental health counsellor, 1 person with an addiction, 1 family caregiver and recipient of home care services)
FG2: Urban Community Health Centre	11 participants (CHC staff with various community roles: chronic disease prevention management, primary care outreach for seniors, nurse practitioner, community health worker)
FG3: Rural CHC	11 participants (1 manager of health services, 2 nurses, 2 nurse practitioners, 2 family physicians, 2 clerks, 1 social worker, 1 respiratory therapist)
FG4: Multicultural Health Navigation	6 participants (navigators who provide services to immigrants and refugees to help them connect to health care providers in their community)
FG5: Members of primary care practice and Community Members	3 participants (patient, family member, addictions counsellor)
FG6: Primary Care Staff	5 participants (1 clinic manager, 2 physicians from a family health team, 1 nurse practitioner and 1 social worker from a community health centre)
**Interview Participants**	
All participants were family physicians; 5 practiced in an urban centre and 1 practiced in a rural community.	

Two of the focus groups were held following completion of the six PCP interviews to further explore community service providers’ perspectives and beliefs about specific aspects of access to care raised in the interviews and prior focus group discussions.

### Data analysis

We developed a coding framework based on the paired dimensions of individuals and system: the characteristics of the health system and service providers; and the capabilities of persons to access services proposed by Levesque et al. [8] We also drew on the attributes of primary care access advanced by Haggerty et al., 2007 and Hogg et al., 2008 [27, 28] to further describe the context. The coding framework identifies the dimension of access, corresponding themes, sub-themes, explanation and examples. An example of one dimension (individual and system level factors) with corresponding theme, sub-theme and explanation can be found in Table 3.

**Table 3:** Coding framework (example of access dimension, themes and sub-themes)

Access Dimension/Category	Theme	Sub-theme	Explanation and Examples.
**1.0 APPROACHABILITY/** **ABILITY TO PERCEIVE** (Levesque, 2013): People facing health needs can actually identify that some form of services exists, can be reached, and have an impact on the health of the individual.	**1.1. Approachability - System Level** (Levesque, 2013): Services can make themselves more or less known among various social or geographical population groups. Various elements such as transparency, information regarding available treatments and services and outreach activities could contribute to make the services more or less approachable.	1.1.1 Transparency	Do primary health care resources make themselves known to providers or the eligible population?
	**1.2 Ability to Perceive - Individual/ Population Level** (Levesque, 2013): The notion of ability to perceive need for care among populations is crucial and determined by such factors such as health literacy, knowledge about health and beliefs related to health and sickness.	1.2.1 Health literacy; technology literacy; knowledge and beliefs about health	Patients’ health beliefs influence their action to prevent illness, reduce severity or symptoms, or have other positive outcomes. Patients’ knowledge of information about their health (e.g., causes, disease processes or progression). Includes patients’ ability to assess and acquire information in a digital environment (use health information technology).

We used a deductive and inductive approach to data analyses. A deductive approach to thematic analysis enabled a rigorous examination of the dimensions of access and their application across the interviews and focus group data to respond to the study objective [29, 30]. An inductive approach helped us to understand the participants’ experience through the identification of themes that emerged from the data and the coders’ interpretation of the text. Prior to independently coding the data, two research assistants (RA) were trained in the theoretical foundation and practical application of the coding framework by a member of the research team involved in its development.

Data analyses was performed by initially listening to the audio-recording of the interviews and focus group data and making notes of categories related to barriers and facilitators to access. Focus groups, in-depth interviews and constant comparison of qualitative analysis by 2 coders and a member of the research team, contributed to the triangulation of data. Each written transcript was then coded by categorizing meaning units using the framework and noting emergent codes. The RAs entered memos to explain their rationale for coding, descriptions about facilitators and barriers to access, and relationships among dimensions of access. Specific quotations that were considered impactful statements or representative of recurring themes were additionally noted. Once the individual coding was complete, a validation process was undertaken involving the RAs and a third member of the research team. Coding of each interview and focus group were discussed and modified as needed to reach consensus about relevant themes and to ensure consistency. Emergent concepts and meanings that were different than the coding framework were noted and discussed. Raters then determined the access domains that were coded most frequently for each interview and focus group. The data were examined from a comparative perspective across the six interviews and six focus groups to confirm the relevant themes. We chose the four most frequently occurring themes as an indication of the importance of the dimension of access.

### Findings

The study findings are presented from the Levesque et al. [8] access framework lens. The most frequent themes involved four dimensions of access: (1) Approachability and Ability to Perceive; (2) Acceptability and Ability to Seek; (3) Availability and Accommodation and (4) Appropriateness. Each of these dimensions included sub-themes: Approachability (information, transparency, outreach); Ability to perceive (health literacy, trust and expectations); Acceptability (patient-provider relationship); Ability to seek (autonomy, culture, personal and social values, health care needs, social support); Availability and accommodation (sustainability); Appropriateness (service integration, information continuity, adequacy and comprehensiveness). Sub-themes that were identified in each of these dimensions can be found in the Focus Group Results (Table 4) and Interview Results (Table 5) with corresponding quotes. Interview data emphasized health system level dimensions whereas focus group data commonly related to the paired dimensions of access involving individual and system level dimensions.

**Table 4:** Focus group themes and sub-themes

Theme	Sub-theme	Focus group participants
**Approachability** The communication used by community resource providers and programs to promote awareness of their services.	Information	“When I was looking for information a lot of it is delivered at a university and only at an information level, not on something practical…[*on an*] application level.” (Mental Health System Clients and Caregivers)
		“If you go on and you regularly find things that are out of date and therefore useless information, you’re going to stop using it quickly.” (Primary Care Staff)
	Transparency	“Well in order for people to use it, they have to know it exists, so it needs to be out there in your face, in different places, all the time and where are those places going to be?” (Rural Community Health Centre)
		“It’s not like we don’t have any services. We have tons of services. The question is people don’t always know where they are or how to get to them.” (Primary Care Staff)
	Outreach	“We do outreach, we’ll go to apartment buildings and… [*meet with clients*] there so try to get rid of that barrier of getting them here [*to the community health centre*].” (Urban Community Health Centre)
		“We did a community health week…so we were all at the mall promoting our services and we were giving coupons to people who wanted to come for a tour of the [*community health*] centre.” (Urban Community Health Centre)
**Ability to Perceive** Individuals identify a need for care, influenced by their capacity to interact with service providers.	Health Literacy	“Because when you have a newcomer, whether they’ve been here for thirty years, which was one of my client’s case, she was here since 1984. And she didn’t have a family doctor. She didn’t know how to navigate the system. Although she understands English, and she speaks English.” (Multicultural Health navigation)
	Trust and Expectations	“If the doctor tells the patient about that certain program, it holds a lot more weight than if, if the nurse does or, you know, or the receptionist does. I mean they believe; they believe in their doctor.” (Primary care staff)
**Acceptability** Elements of the patient-provider relationship comprise interpersonal communication, respect and sensitivity.	Patient-Provider Relationship	“For most people they need someone very approachable as the first place of contact when they phone one of these places.” (Primary care staff)
**Ability to Seek** Individuals’ capacity to seek care.	Autonomy	“If you’ve got a mental health issue or you’re struggling with an addiction issue…these people are not equipped to be able to do that kind of searching on their own, and [*are*] easily frustrated.” (Mental Health Systems Clients and caregivers)
		“To educate [*people with* addictions] on their own illness, to make them aware enough so that …they can present themselves in a way that leaves them with their dignity and demands to be treated properly. So we’re now trying to teach them how to be people accessing services that can demand access and demand respect.” (Mental Health Clients and Caregivers)
	Culture	“I find like a lot of my community, as we have issues with language and literacy, so an idea of making an appointment to see a doctor is a very new thing. They’d rather go to a walk-in.” (Multicultural Health Navigators)
		“There’s different concepts of time and we’ve been told that in some parts of either northern Kenya and southern Somalia there used to be time differences so when you say the appointment is at 9:10 they might think, okay I can come anytime from 9:00 to 10:00.” (Urban Community Health Centre)
	Personal and Social Values	“Being able to actually get a family doctor and I think there’s a lot of prejudice around addiction specifically so people with addictions… just have difficulty accessing health care period, because there is a stigma that’s attached to that.” (Mental Health System Clients and Caregivers)
		“When they say, ‘I don’t want to go to this counsellor because they speak my language, they might know me, I don’t want to go back.’ Like I had a client who was struggling with alcoholism. I couldn’t send him back to the Nepali speaking counsellor because he didn’t want to go.” (Urban Community Health Centre)
	Health Care Needs	“I know in the last few years there’s been a lot of focus on teenagers’ mental health, but everything else seems to be forgotten about now… there’s still the rest of the population that has anxieties and problems. And there aren’t enough services for them.” (Mental Health System Clients and Caregivers)
	Social Support	“We do a lot of advocating. We do sometimes have to get in there and help the patient …do the talking, get the right language, get the right service, get the right things happening for the patient.” (Rural Community Health Centre)
		“We [*as a health navigator*] we’re with the client, not to make them depend on us for everything…we’re doing this to empower them and helping them to pass the transition and to say, you can do it yourself.” (Multicultural Health Navigation)
		“If it’s anxiety for instance, [*the client*] may not really discuss the anxiety but they might be able to discuss the fact that they need someone to accompany them.” (Community members)
**Availability and Accommodation** The existence of resources to meet patient needs including geographic location, physical accessibility of the environment in which services are offered, and the methods of program delivery (e.g. in-person, virtual appointments).	Sustainability	“There are good services in the city and often they don’t get any, financial support whatsoever and or funding and …they’re always about to be axed. There’s nothing more aggravating than the best services in the city are being forced to shut their doors because they can’t get any funding.” (Mental Health System Clients and Caregivers)

**Table 5:** PCP interview themes and sub-themes

Themes	Sub-theme	PCP participants
**Approachability** The communication used by community resource providers and programs to promote awareness of their services.	Transparency	“I really like that idea of having this information [about health programs] in the waiting room for people to initiate the referral themselves.” (Primary care provider 04)
		“There’s a huge knowledge deficit…people out there probably feel they’ve got wonderful working programs, but unless I’m physically aware of it, it means nothing.” (Primary care provider 01)
	Information	“One of the hardest things for a new [medical school] graduate is to know those services that are available if there’s no obvious place to go look for it.” (Primary care provider 02)
		“Some sort of dedicated web site that [is] maintained actively and well promoted, that any community agency that wanted…could be found on this site and one site only.” (Primary care provider 02)
**Availability** The existence of resources to meet patient needs including geographic location, physical accessibility of the environment in which services are offered, and the methods of program delivery (e.g. in-person, virtual appointments).	Health care needs	“I only see patients three half days a week. I have maybe thirty appointments available per week…I have nine hundred patients, essentially. There’s no time to bring [a patient] in once a week or once every two weeks [for routine care].” (Primary care provider 03)
		“And why don’t I think I do the follow up [with the patients]? Because I basically have too many competing demands.” (Primary care provider 05)
**Appropriateness** The alignment between individuals’ needs and characteristics of the community resources.	Service Integration (Referral Processes)	“I create a referral in my electronic medical record. We have a database [with] the various specialists that we commonly use…when that electronic consultation is created then my staff prints it off and faxes it to the specialist that I’ve attached to the referral." (Primary care provider 02)
		“Our clinic refuses to even do forms, like we put everything into the EMR…Very rarely do we actually submit a paper form because it becomes so time consuming with our EMR and then it’s not as readily accessible for us.” (Primary care provider 04)
	Information Continuity	“I don’t need to know huge details unless there are some concerns and we need to collaborate [with the community resource] around it, but it would be nice to know about what services [patients are] accessing.” (Primary care provider 04)
		“I can almost picture developing some kind of standardized feedback form from all these different providers…where the top half might just be patient attended this many visits, this is the scope of what they learned, right.” (Primary care provider 06)
	Adequacy/Comprehensiveness	“It seems burdensome for [patients] and it’s because all the services are separated. It’s overwhelming for them to try and access multiple types of services. But if there’s one navigator that helps them kind of triage what’s important, where they can get the most bang for their buck.” (Primary care provider 04)
		“Navigators are about making the connections and knowing enough to make the best possible connection for the patient at that time. But they’re not really meant to provide care in any way themselves.” (Primary care provider 06)

### Approachability and ability to perceive

#### Promotion of health enabling community resources

PCPs expressed that while there are many useful resources available, organizations did not promote their services to them or to the eligible patient population. This limited PCP referrals to HERs and patients’ ability to perceive and access these resources. PCP and focus group participants suggested strategies such as placing pamphlets in practice waiting rooms, pharmacies, libraries, and other community locations to increase the visibility of services and programs. One PCP mentioned that promotional material in their waiting room would foster patient awareness enabling them to potentially contact the resource directly. Another PCP proposed that existing information directories of community resources should be better promoted to PCPs and patients. Participants who were aware of online directories of resources said these were difficult to navigate due to the complexity and large volume of information. Resource and program details should be up-to-date and communicated through user-friendly, practical mediums for patients that are easy to navigate and at an appropriate literacy level to facilitate access.

PCPs expressed that they required more information about the available HERs in their community to feel better equipped to support the needs of their patients and refer them to the appropriate resource. An FGD respondent noted: “I think lack of knowledge is the thing that probably challenges me the most, not being aware of what’s available therefore I can’t give the information out.” (Primary Care Staff) They preferred information from the internet to facilitate quick access and expressed the need for one centralized and comprehensive online information directory with relevant information about a variety of HERs in their region. An interview respondent noted, “I think some level of improved promotion of that [directory of community resources] would be really beneficial for all providers so that it could sort of expedite the searching time.” (Primary care provider 02).

### Time as a limited health care resource

PCPs also identified that it was a challenge to search for HERs due to limited time allocated for patient appointments and a high patient caseload. On average a physician patient appointment is scheduled under 10–15-minute time intervals. These factors also constrained their ability to follow-up with patients about the outcome of a referral to a community resource. Some PCPs expressed feeling overwhelmed by taking on additional activities that were navigational in nature and time-consuming, such as searching through directories to find an appropriate resource for a patient considering factors such as eligibility criteria, location of the service, and associated fees. They were unprepared to assume these activities and felt that these could be addressed more efficiently by a social worker or navigator, who were knowledgeable about local community resources.

### Navigation of health enabling community resources

Participants suggested that PCPs and community health centre staff be trained on how to navigate online resource directories. Mental Health focus group participants specifically noted that written information is preferable for persons who may not have access to a telephone or a computer, or for patients with anxiety or other mental health problems that may interfere with their ability to retain verbal information. The Rural Community Health Centre focus group expressed that programs should provide outreach and communication strategies to reach rural populations such as advertising in community newspapers for older adults.

Patients face barriers such as lack of awareness and knowledge about how resources may be beneficial for their health A low level of health literacy and a lack of trust or a negative view of the health care system were recurrent barriers identified in the focus groups. Newcomers or immigrants experience challenges in learning how to navigate the health care system, such as making an appointment with a primary care provider. Participants identified that an interpreter or a patient navigator can facilitate access by connecting or accompanying the patient to needed services. Trust was an important factor influencing ability to perceive a need for care particularly among patients with mental health problems or addictions. Like one respondent noted: “Because my son has had a lot of negative interactions with health professionals, I can’t even get him there for a checkup.” (Mental Health System Clients and Caregivers). In the Mental Health focus group, participants also discussed the value of ‘word of mouth’ in learning about the quality of programs and the eligibility criteria for access and participation in HERs.

### Acceptability and ability to seek

#### Patient-service provider relationship

Patient trust was an element influencing acceptability and ability to seek care. Primary Care Staff and Multicultural Health Navigator focus group participants identified that a positive patient-provider relationship creates an environment of trust that allows the discussion of difficult health concerns, facilitating the access dimension of acceptability. Another dimension related to trust and access to care concerned the context of services. A PCP explained that patients were more likely to attend a chronic pain clinic offered at their practice, a known and comfortable setting, rather than in an unfamiliar context. Participants also noted that patients’ first point of contact with a PCP or a community resource should be met by acceptance and non-judgmental listening to create a safe environment and allow them to talk openly and share information about themselves. Focus group participants also expressed that a relationship founded upon respect allows culturally sensitive issues such as aging or personal hygiene to be discussed. A climate of acceptability was particularly important to persons with cognitive impairment, those who were socially isolated, and persons with mental illness who experience stigma as a barrier to seeking care. One of the respondents noted, “A lot of the vulnerable sector you really need to develop that relationship before they accept any services and be accepting of where they are in their life, right. And that’s really important, knowing where they are and where they want to go and then helping them get from A to B.” (Urban Community Health Centre).

Youth were identified as a particular population that struggled in perceiving and seeking HERs and engaging with providers. Additional support was recommended to help youth navigate the complexities of access to HERs such as adopting a family-centred approach to enable follow through of PCP recommendations. Other factors, including the individual’s social and cultural context, personal values, physical or cognitive limitations, may present barriers to seeking care. The Multicultural Health Navigator focus group shared how a new immigrant family that required a speech language program for their child, could not access the service because they did not speak English and did not understand how to access this therapy. Culturally appropriate services to overcome language barriers were recommended to facilitate patients’ ability to seek HERs.

### Availability and accommodation

#### Meeting patient needs

PCPs and focus group participants differed in their perception of this access dimension. PCPs commonly noted limited availability of resources to meet patient needs as a barrier to access, whereas focus group participants identified lack of accommodation by HERs and primary care providers as a barrier. Mental Health and Community Member focus group participants expressed that they often had insufficient time to meet with their PCP to discuss illness prevention and health behavior needs and resources. They felt rushed and anxious in a short appointment, and this affected their ability to process the information they received from their PCP about their health. Persons with addictions or who had experienced sexual abuse were identified as particularly vulnerable in a time constrained provider encounter. One of the respondent noted, “I’ve had ten traumas and so I need to have a lot of extra support around seeing someone and if it’s a tight time frame, which is often with the family physicians…the manner is anxiety-producing…they don’t have time to process or give information…” (Mental Health System Clients and Caregivers). A primary care provider expressed in the interview, “I find myself sometimes thrown into a social worker type role that I’m completely unprepared to do. I don’t have the knowledge. I don’t have resources for it, and frankly it’s a time-consuming thing to be looking into all the various resources that a social worker would already probably know off the top of their head.” (Primary care provider 04).

### Funding and operating hours

Participants expressed frustration at the closure of a number of helpful community-based mental health resources, leading to increased reliance on hospital-based care. The focus group participants also identified limited appointment flexibility and operating hours, constraints on the duration of services and number of sessions offered, and reduced program funding as barriers to reach HERs. For participants in the Mental Health focus group, insufficient government funding for organizations offering mental health care or a loss of funding meant reduced availability of services. Community members expressed the need for mental health resources specific to certain populations such as those with a personality disorder or hoarding disorder. While participants acknowledged there was a positive recent focus on support for youth mental health, they also expressed the inadequate availability of services for seniors and the specific mental health issues they face.

### Appropriateness

#### Referrals and communication

This theme was identified solely in the PCP interview data. Reoccurring subthemes under the dimension of appropriateness included service integration, continuity of information and comprehensiveness of care relating to the navigator. The use of paper forms and/or the electronic medical record (EMR) to refer patients to HERs varied across practices and PCPs. Those who used the EMR as their primary method of referral stated that completing paper forms was time consuming and forms were not readily available. For others, paper referrals were sent by fax to community resources, as they felt that modes of communication such as email were not secure.

PCPs reported that the channels of communication with the HERs was inadequate, including a paucity of information about their patients and the outcome of accessing a community program and service. They recommended a standard patient feedback form from the community provider that would allow the PCP to deliver better follow up care.

### Patient navigation

Finally, PCPs expressed the need for comprehensive patient navigation services and viewed this as an important asset to their practice. They recommended that a navigator have a non-clinical role and be knowledgeable about guiding patients through the complexities of the community health and social system. One PCP described this role as making the best possible connection with a community resource for a patient at a given time. A navigator would also assist in reducing providers’ workload by relieving them from the time-consuming process of finding appropriate resources and helping patients connect to the most appropriate service based on their individual needs and context. A recurring suggestion among PCPs was that navigators should work within the primary care practice, as patients are comfortable coming to the practice and would be more likely to access the navigator in a familiar setting. One of the interview respondents noted, “I think it would be nice to co-locate [the navigator] in the practice. For those patients in particular, they’re much more likely to access the navigators.” “I can get him [a patient] on the road to thinking about it, but I can’t do it for him. And he’s got to want to do it for himself as well, but he needs a guide. And in this case, that’s a navigator.” (Primary care provider 03). Although most PCPs felt their practice could benefit from introducing a navigator, one provider was hesitant to the navigator approach, feeling it was the PCP’s role and responsibility to know what resources are available to patients and to connect patients with the appropriate resources.

## Discussion

In this study, Primary care physicians and community stakeholders shared their perspectives and experiences about barriers and facilitators to accessing resources in the community. Participants provided a unique viewpoint to understand access at a client, practice, and local level. The primary access themes identified in our findings helped to inform the ARC navigation model and corresponding intervention activities. These will be described in the study implications section.

PCPs discussed challenges in addressing the social determinants of health for their patients and possible solutions to inequitable access. The interviews revealed that approachability, availability, and appropriateness of community resources at a system level, significantly impacted patient access to care. Community-based organizations need to be approachable to providers and patients to be utilized. PCPs desired information that is relevant and practical, to enable them to effectively act on patients’ health and social needs [31, 32]. It is recommended that organizations maintain and promote complete and current information about their services including the location, hours of operation, eligibility criteria, appointment mechanisms, and associated fees. A user-friendly digital repository of information about community resources [33] can help to reduce barriers to approachability and ability to perceive. Participants advised that information should be indexed to priority health topics such as mental health, to facilitate the finding of relevant resources.

Adequate literacy levels are a large predictor of individuals’ ability to understand and navigate the health care system, which includes being able to act on PCP referrals and reach community resources [34, 35]. Patients require information that is current and relevant, and written in language that meets health literacy standards to enable an understanding of the material. Programs that provide health information online should be easy for patients to use and navigate to prevent additional barriers related to digital literacy.

Consideration is also needed for outreach initiatives to vulnerable groups that may not have access to the computer, internet, or a primary care provider. Support groups such as those for addictions and mental health, can be a venue to promote awareness of available resources for health and social needs. It is challenging for PCPs to remain up to date with available community resources for their patients, as found in previous studies [15, 20]. Participants recommended training PCPs on how to access information on local HERs and embedding a referral process in the electronic medical record, as strategies to increase providers’ knowledge about available community programs, promote referral, and track patients’ outcomes related to HERs. PCPs also recommended increasing the transparency of HERs by placing informational pamphlets in a primary care practice waiting room to promote awareness among patients.

Focus group participants identified that culture and language barriers influence individuals’ ability to seek and access community resources. Language discordance extends beyond the patient-provider interaction and may impact quality of care and patients’ ability to access services for health and well-being needs [36]. Currently, Ontario health care plans do not cover translation services, and cultural interpretation is allocated to poorly funded community health centres. Finally, participants identified that access to health care services can be facilitated when operating hours accommodate the needs of its target population (e.g. being open on weekends and weeknights to accommodate persons that are working or providing child care or elder care).

Participants’ discussions surrounding the potential scope and role of a navigator helped provide insight into the ARC navigation model and how it could be embedded in a primary care setting. At a provider level, a navigator may promote awareness of available HERs for their patient needs. This would help to address the concerns of many PCPs who identified the challenge of being knowledgeable about resources to address their patients’ social determinants of health. The findings suggest that navigators need to be individuals with the knowledge and skills to support and connect patients to resources, allowing providers to devote their limited time to other patient priorities. Study participants emphasized that navigators should assume non-clinical roles in which they do not offer medical advice or care to patients. At a patient level, a navigator situated in primary care, could help foster acceptability in an environment that they trust [37]. A navigator could help identify appropriate HERs to meet individuals’ needs and priorities, assist in overcoming barriers such as location of the service, transportation, and funding, and provide emotional and social support to facilitate access [38]. Patient navigation is conceived as a person-centered approach to empower individuals, promote patient engagement, and address health disparities [39–41]. In summary, navigation services have the potential to support continuity of primary and community care, and increase approachability, availability, and accommodation as well as appropriateness of community resources.

### Study implications

The conceptualization of access based on the framework of Levesque et al. [8] enabled an understanding of the facilitators and barriers to access care from the perspectives of PCPs and community stakeholders. Participants’ perspectives about the potential scope and role of a patient navigator provided valuable insight for the development of the ARC as a de novo navigation model and how it could be embedded in a primary care setting. Based on the findings, the following innovations will be leveraged to facilitate access to HERs for primary care patients in the ARC study:

1) PCPs and their staff will be oriented to the breadth of available health and social resources to which they can refer their patients. Clinic processes that fit into the routines of daily practice will be developed to support providers to refer to these resources including a standardized referral form to identify patient needs and navigation services to support patients’ access to resources.

2) Available community resources will be promoted to patients in the practice waiting room through informational brochures and videos. Patients will also be taught how to use existing online and telephone directory of information services [33] so that they are empowered to identify and access needed resources.

3) A lay patient navigator will be integrated within the primary care practice to help patients address and overcome barriers to access. The navigator will serve as a link between patients, primary care, and community care through collaboration and effective exchange of information and leverage community resources to address patients’ social determinants of health.

### Study limitations

We advance four limitations to this study: (1) The PCPs participants were recruited from a similar demographic; those practicing in an urban setting and serving a patient population that were predominantly middle to high socioeconomic status. (2) The access dimension of Affordability and Ability to Pay did not emerge as primary barriers. The five dimensions of access as defined by Levesque et al. [8], can be thought of as a continuum that ranges from the early stages of access to the later stages of access. Focus group members reported barriers to the earlier stages of access much more frequently than the later stages. Our assumption is that if patients cannot successfully perceive, seek, and reach community resources, they may have not yet been faced with barriers concerning services’ affordability. Another potential explanation is that the study was conducted in a resource-rich region with many low costs or no cost community health and social resources. Data collection methods, participants, and/or patient population served, may also have contributed to this finding. (3) The composition of the focus groups and the nature of the PCPs’ patient population may have not reflected the social complexity seen in primary care patients. The study was a single center study, interviewing PCPs only in one health region, whose experiences may not be representative of other PCPs or other primary care providers such as nurses, nurse practitioners or social workers. Increasing the sample size and diversity in terms of physician demographics, and the patient populations served (socioeconomic areas, geographic regions) would enable a broader understanding of the challenges in caring for primary care patients with social barriers and how to support them in accessing community-based primary health care. A comparison with other IMPACT sties was not feasible as each IMPACT sites had different priority. (4) Finally, the robustness of the study is limited using purposive sampling to recruit interview and focus group participants. Further consultation with key stakeholders in the health region including patients, primary care providers, community service providers and health planners was therefore undertaken to inform the design of the ARC intervention.

## Conclusion

In conclusion, primary care offers a context to address the social determinants of health and access to care through patient navigation. Navigators may bridge a critical gap at the system and individual levels that prevent access to community resources due to challenges in approachability, ability to perceive, acceptability, ability to seek, availability and accommodation.

### Electronic supplementary material

Below is the link to the electronic supplementary material.

Supplementary Material 1

## Data Availability

Supplementary material is provided with the manuscript. This includes the study tools (i) Appendix A: Focus Group Discussion guide and (ii) Appendix B: Interview guide.

## Abbreviations

IMPACT: Innovative Models Promoting Access-to-Care Transformation
HERs: Health Enabling Community Resources
ARC: Access to Resources in the Community
PCPs: Primary Care Providers
RA: Research Assistants
EMR: Electronic Medical Record
