# Supplementary material for: Access to primary health care: perspectives of primary care physicians and community stakeholders
Source: BMC Prim Care. 2024 May 6;25:152. doi: 10.1186/s12875-024-02312-9 (PMC11071188; doi:10.1186/s12875-024-02312-9)
Supplement: Supplementary file 1 — Supplementary Material 1 [file 12875_2024_2312_MOESM1_ESM.docx]

# Supplementary Material

# Appendix A: Focus Group Discussion Guide

**Understanding Existing Supports**

1. Can you tell us about your organisation? (e.g. aims, programs/services, population served)
2. Can you tell us about the type of support you usually provide to your clients or community members to access needed primary care related to and/or community-based programs that support health?
3. What considerations/accommodations are already made to increase access to needed care, programs, services at your Centre?
4. What changes could be made at your Centre to increase access to needed care for your clients or community members?
5. What programs/services are not available at your Centre that you have connected your clients or community members with in the past to help address a health issue or issue that impacts their health?

**Looking for Information**

1. How did you look for information about programs/services not available at your Centre that support health? What information resources do you use? (e.g., Google, brochures, colleagues)
2. When you use the resources you have described, do you usually find the information that you are looking for? If yes, what makes these resources easy to use/access? If no, what makes these resources difficult/challenging to use/access?
3. Thinking broadly, what would make it easier for you or your clients/community members who face the biggest barriers to accessing needed care to find the care they need at community programs?

We are exploring ways to increase access for primary care providers to find the information needed to help connect their clients/community members with available programs. This may involve initiatives aimed at providers and other practice staff to train them on how to connect their vulnerable clients with community programs using resources like Champlain Health Line. The intervention may also include initiatives to increase communities’ awareness of these resources.

1. Do you think that this approach would be helpful for you or your clients or community members to be more aware of these resources? Why or why not?
2. How do you think that we could help to ensure that such an initiative would lead to improved access to care for individuals facing barriers?
3. Are there aspects of this proposed intervention that would make it more difficult for your clients/community members to access care?

# Appendix B: Interview Guide for Primary Care Physicians

**Referral to Community Resources/Access Barriers**

1. What are the most common social barriers limiting your patients from obtaining the care they need?
2. How do you currently refer to other health services? (specialists, allied health professionals etc.)
3. How do you currently connect patients to community resources like diabetes education, self-management support, or other programs in the community to address their health needs?
4. Would a systematic process for recommending community resources be acceptable to build into your usual care? a) Would you use a referral form? b) Would it be feasible to embed this in your EMR?

**Navigation**

1. Would it be feasible/acceptable in your practice to have you or a member of your staff assist with helping patients to reach a community resource by calling 211 on their behalf, or arranging transportation or language interpretation services?
2. Would it be feasible/acceptable in your practice to have a patient navigator (implemented by our team) to assist patients with complex social barriers to reach a community resource?
3. How would you like to receive information from the navigator about the programs/services accessed by your patient?
